# Tribenzylbis(triphenyl­arsine oxide-κ*O*)tin(IV) tetra­phenyl­borate

**DOI:** 10.1107/S1600536810039450

**Published:** 2010-10-09

**Authors:** Thy Chun Keng, Kong Mun Lo, Seik Weng Ng

**Affiliations:** a Department of Chemistry, University of Malaya, 50603 Kuala Lumpur, Malaysia

## Abstract

The crystal structure of the title salt, [Sn(C_7_H_7_)_3_(C_18_H_15_AsO)_2_][B(C_6_H_5_)_4_], consists of discrete cations and anions; the tin atom of the cation is five-coordinated in a distorted *trans*-C_3_SnO_2_ trigonal-bipyramidal geometry [summation of C–Sn–C angles 360.0 (3)° and O–Sn–O angle 173.1 (1)°]. The structure contains voids of 113 (19) Å^3^, but no solvent mol­ecule could reasonably be located there.

## Related literature

For the structure of a related triorganotin tetra­phenyl­borate, see: Ng *et al.* (1989 ▶). 
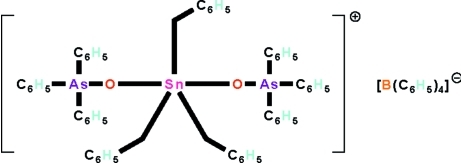


## Experimental

### 

#### Crystal data

[Sn(C_7_H_7_)_3_(C_18_H_15_AsO)_2_](C_24_H_20_B)
*M* *_r_* = 1355.72
Triclinic, 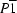

*a* = 14.3288 (7) Å
*b* = 14.9477 (7) Å
*c* = 19.0994 (9) Å
α = 69.220 (1)°
β = 73.972 (1)°
γ = 62.683 (1)°
*V* = 3366.4 (3) Å^3^
*Z* = 2
Mo *K*α radiation
μ = 1.40 mm^−1^
*T* = 100 K
0.1 × 0.1 × 0.1 mm

#### Data collection

Bruker SMART APEX diffractometer
Absorption correction: multi-scan (*TWINABS*; Bruker, 2009 ▶) *T*_min_ = 0.788, *T*_max_ = 1.000
76329 measured reflections
17932 independent reflections
12383 reflections with *I* > 2σ(*I*)
*R*_int_ = 0.068

#### Refinement

*R*[*F* ^2^ > 2σ(*F* ^2^)] = 0.050
*wR*(*F* ^2^) = 0.170
*S* = 0.99
17932 reflections
629 parameters
H-atom parameters constrained
Δρ_max_ = 2.06 e Å^−3^
Δρ_min_ = −1.10 e Å^−3^

### 

Data collection: *APEX2* (Bruker, 2009 ▶); cell refinement: *SAINT* (Bruker, 2009 ▶); data reduction: *SAINT*; program(s) used to solve structure: *SHELXS97* (Sheldrick, 2008 ▶); program(s) used to refine structure: *SHELXL97* (Sheldrick, 2008 ▶); molecular graphics: *X-SEED* (Barbour, 2001 ▶); software used to prepare material for publication: *publCIF* (Westrip, 2010 ▶).

## Supplementary Material

Crystal structure: contains datablocks global, I. DOI: 10.1107/S1600536810039450/xu5034sup1.cif

Structure factors: contains datablocks I. DOI: 10.1107/S1600536810039450/xu5034Isup2.hkl

Additional supplementary materials:  crystallographic information; 3D view; checkCIF report

## Figures and Tables

**Table:** 

Sn1—C1	2.150 (5)
Sn1—C8	2.145 (5)
Sn1—C15	2.144 (5)
Sn1—O1	2.175 (3)
Sn1—O2	2.225 (3)

**Table:** 

C1—Sn1—C8	114.4 (2)
C1—Sn1—C15	119.7 (2)
C8—Sn1—C15	125.9 (2)
O1—Sn1—O2	173.1 (1)
As1—O1—Sn1	165.3 (2)
As2—O2—Sn1	160.3 (2)

**Fig. 1.**
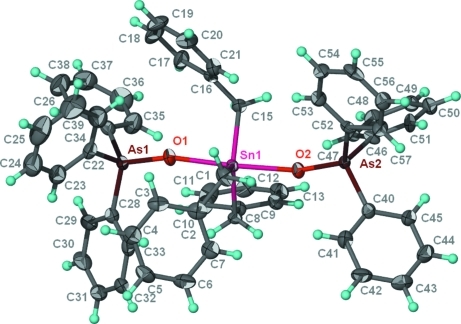
Thermal ellipsoid plot (Barbour, 2001) of the cation of [Sn(C7H7)3(C18H15AsO)2] [B(C6H5)4] at the 70% probability level; hydrogen atoms are drawn as spheres of arbitrary radius.

**Fig. 2.**
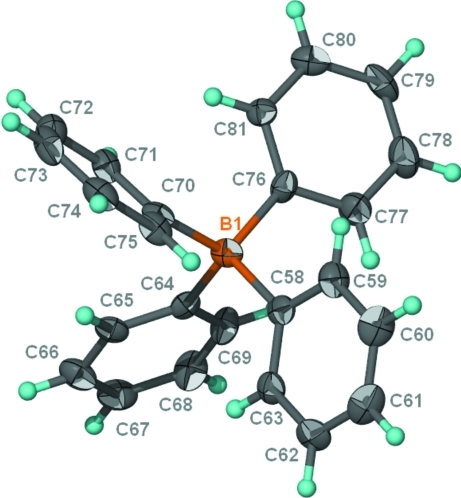
Thermal ellipsoid plot (Barbour, 2001) of the anion of [Sn(C7H7)3(C18H15AsO)2] [B(C6H5)4] at the 70% probability level; hydrogen atoms are drawn as spheres of arbitrary radius.

## supplementary crystallographic information

### Comment

A suggestion for improving the aqueous solubility of triorganotin(IV) compounds, *R*_3_SnX, involves exchanging the *X* residue by the non-nucleophilic tetraphenylborate anion to generate a salt. However, to prevent aggregation into an oligopolymer, the triorganotin species itself must be stabilized by coordination by two neutral ligands. Of the number of such compounds synthesized, the crystal structure of only one, namely, [(*p*-ClC_6_H_4_)Ph_2_Sn(Ph_3_AsO)_2_][BPh_4_], has been reported (Ng *et al.*, 1989). The compound exists as non-interacting cations and anions; the tin atom of the cation is five-coordinate in a *trans*-C_3_SnO_2_ trigonal bipyramidal geometry. The title tribenzyltin adduct (Scheme I, Figs. 1 & 2) is a similar non-interacting salt. The *trans* angle at tin is nearly linear, and the Sn–O–As skeleton is also nearly linear (Table 1).

### Experimental

Tribenzyltin chloride (0.21 g, 0.5 mmol), triphenylarsine oxide (0.32 g, 1 mmol) and sodium tetraphenylborate (0.17 g, 0.5 mmol) were dissolved in ethanol (100 ml). The solution was heated for an hour. Then the mixture was filtered and the filtrate evaporated at room temperature to obtain the colorless crystals.

### Refinement

Carbon-bound H-atoms were placed in calculated positions (C—H 0.95 Å) and were included in the refinement in the riding model approximation, with *U*(H) set to 1.2*U*(C). All aromatic rings were refined as rigid hexagons of 1.39 Å sides.

The final difference Fourier map had a high peak at 3.22 Å from H39 and hole at 0.83 Å from Sn1. The peak could not be refined as a water O atom, and is probably an artifical of twinning. The structure contains a voids of 113 (19) Å^3^, but no solvent molecule(s) could be located there reasonably.

### Crystal data

**Table:**

[Sn(C_7_H_7_)_3_(C_18_H_15_AsO)](C_24_H_20_B)	*Z* = 2
*M**_r_* = 1355.72	*F*(000) = 1388
Triclinic, *P*1	*D*_x_ = 1.337 Mg m^−^^3^
Hall symbol: -P 1	Mo *K*α radiation, λ = 0.71073 Å
*a* = 14.3288 (7) Å	Cell parameters from 4476 reflections
*b* = 14.9477 (7) Å	θ = 2.3–28.2°
*c* = 19.0994 (9) Å	µ = 1.40 mm^−^^1^
α = 69.220 (1)°	*T* = 100 K
β = 73.972 (1)°	Cube, colorless
γ = 62.683 (1)°	0.1 × 0.1 × 0.1 mm
*V* = 3366.4 (3) Å^3^	

### Data collection

**Table:**

Bruker SMART APEX diffractometer	17932 independent reflections
Radiation source: fine-focus sealed tube	12383 reflections with *I* > 2σ(*I*)
graphite	*R*_int_ = 0.068
ω scans	θ_max_ = 27.5°, θ_min_ = 1.2°
Absorption correction: multi-scan (TWINABS; Bruker, 2009)	*h* = −17→18
*T*_min_ = 0.788, *T*_max_ = 1.000	*k* = −17→19
76329 measured reflections	*l* = 0→24

### Refinement

**Table:**

Refinement on *F*^2^	Primary atom site location: structure-invariant direct methods
Least-squares matrix: full	Secondary atom site location: difference Fourier map
*R*[*F*^2^ > 2σ(*F*^2^)] = 0.050	Hydrogen site location: inferred from neighbouring sites
*wR*(*F*^2^) = 0.170	H-atom parameters constrained
*S* = 0.99	*w* = 1/[σ^2^(*F*_o_^2^) + (0.1059*P*)^2^] where *P* = (*F*_o_^2^ + 2*F*_c_^2^)/3
17932 reflections	(Δ/σ)_max_ = 0.001
629 parameters	Δρ_max_ = 2.06 e Å^−^^3^
0 restraints	Δρ_min_ = −1.10 e Å^−^^3^

### Fractional atomic coordinates and isotropic or equivalent isotropic displacement parameters (Å^2^)

**Table:**

	*x*	*y*	*z*	*U*_iso_*/*U*_eq_	
Sn1	0.68359 (3)	0.68691 (2)	0.973010 (17)	0.01457 (9)	
As1	0.58668 (4)	0.62392 (4)	1.18383 (3)	0.01939 (13)	
As2	0.77629 (4)	0.73481 (4)	0.76041 (3)	0.01624 (12)	
O1	0.6109 (3)	0.6565 (3)	1.09025 (18)	0.0275 (9)	
O2	0.7611 (3)	0.6987 (3)	0.85374 (18)	0.0239 (8)	
B1	0.8611 (5)	0.8755 (5)	0.3893 (3)	0.0213 (12)	
C1	0.6220 (5)	0.8523 (4)	0.9593 (3)	0.0271 (12)	
H1A	0.6472	0.8862	0.9075	0.033*	
H1B	0.5436	0.8813	0.9652	0.033*	
C2	0.6556 (3)	0.8774 (3)	1.01589 (17)	0.0260 (12)	
C3	0.5848 (2)	0.9025 (3)	1.07932 (19)	0.0261 (12)	
H3	0.5151	0.9059	1.0860	0.031*	
C4	0.6159 (2)	0.9228 (3)	1.13289 (16)	0.0298 (13)	
H4	0.5675	0.9399	1.1762	0.036*	
C5	0.7177 (3)	0.9179 (3)	1.12305 (18)	0.0300 (13)	
H5	0.7390	0.9317	1.1597	0.036*	
C6	0.7885 (2)	0.8927 (3)	1.0596 (2)	0.0324 (13)	
H6	0.8582	0.8894	1.0529	0.039*	
C7	0.7574 (2)	0.8725 (3)	1.00605 (16)	0.0283 (12)	
H7	0.8058	0.8553	0.9627	0.034*	
C8	0.8415 (4)	0.6017 (4)	1.0023 (3)	0.0193 (10)	
H8A	0.8903	0.6274	0.9613	0.023*	
H8B	0.8434	0.6150	1.0489	0.023*	
C9	0.8804 (3)	0.48387 (18)	1.01481 (17)	0.0211 (11)	
C10	0.8659 (3)	0.4197 (2)	1.08652 (14)	0.0295 (13)	
H10	0.8347	0.4484	1.1287	0.035*	
C11	0.8970 (3)	0.3137 (2)	1.09650 (15)	0.0364 (14)	
H11	0.8871	0.2698	1.1455	0.044*	
C12	0.9425 (3)	0.27177 (18)	1.0348 (2)	0.0321 (13)	
H12	0.9638	0.1993	1.0416	0.038*	
C13	0.9570 (3)	0.3359 (2)	0.96306 (16)	0.0270 (12)	
H13	0.9882	0.3073	0.9209	0.032*	
C14	0.9259 (3)	0.4420 (2)	0.95308 (13)	0.0233 (11)	
H14	0.9358	0.4858	0.9041	0.028*	
C15	0.5900 (4)	0.6224 (4)	0.9532 (3)	0.0204 (10)	
H15A	0.5939	0.6364	0.8980	0.024*	
H15B	0.6208	0.5457	0.9747	0.024*	
C16	0.4742 (2)	0.6659 (3)	0.98714 (18)	0.0210 (11)	
C17	0.4353 (3)	0.6003 (2)	1.04697 (19)	0.0319 (13)	
H17	0.4809	0.5295	1.0660	0.038*	
C18	0.3298 (3)	0.6384 (3)	1.07905 (18)	0.0447 (17)	
H18	0.3032	0.5935	1.1199	0.054*	
C19	0.2631 (2)	0.7420 (3)	1.0513 (2)	0.0452 (17)	
H19	0.1910	0.7680	1.0732	0.054*	
C20	0.3020 (2)	0.8076 (2)	0.9914 (2)	0.0382 (15)	
H20	0.2564	0.8784	0.9725	0.046*	
C21	0.4075 (3)	0.7696 (2)	0.95937 (16)	0.0302 (13)	
H21	0.4341	0.8144	0.9185	0.036*	
C22	0.4714 (2)	0.7369 (3)	1.21711 (19)	0.0233 (11)	
C23	0.4759 (3)	0.7645 (3)	1.2780 (2)	0.0311 (13)	
H23	0.5379	0.7284	1.3021	0.037*	
C24	0.3896 (3)	0.8448 (3)	1.3037 (2)	0.0439 (16)	
H24	0.3926	0.8637	1.3453	0.053*	
C25	0.2988 (3)	0.8977 (3)	1.2684 (2)	0.054 (2)	
H25	0.2398	0.9526	1.2860	0.065*	
C26	0.2943 (3)	0.8701 (3)	1.2075 (2)	0.0545 (19)	
H26	0.2322	0.9062	1.1835	0.065*	
C27	0.3806 (3)	0.7897 (3)	1.18188 (19)	0.0414 (15)	
H27	0.3775	0.7709	1.1403	0.050*	
C28	0.7089 (2)	0.5866 (3)	1.22728 (17)	0.0220 (11)	
C29	0.7405 (3)	0.4986 (2)	1.28777 (18)	0.0271 (12)	
H29	0.7003	0.4571	1.3086	0.032*	
C30	0.8308 (3)	0.4715 (2)	1.31782 (16)	0.0334 (14)	
H30	0.8523	0.4114	1.3592	0.040*	
C31	0.8896 (2)	0.5323 (3)	1.28739 (19)	0.0302 (13)	
H31	0.9513	0.5138	1.3079	0.036*	
C32	0.8581 (3)	0.6202 (3)	1.22690 (18)	0.0297 (13)	
H32	0.8983	0.6618	1.2061	0.036*	
C33	0.7678 (3)	0.6474 (2)	1.19685 (15)	0.0236 (11)	
H33	0.7462	0.7075	1.1555	0.028*	
C34	0.5484 (3)	0.5070 (2)	1.22101 (19)	0.0230 (11)	
C35	0.6048 (3)	0.4222 (3)	1.19022 (19)	0.0374 (14)	
H35	0.6599	0.4241	1.1491	0.045*	
C36	0.5808 (3)	0.3347 (2)	1.2196 (2)	0.0441 (16)	
H36	0.6194	0.2767	1.1986	0.053*	
C37	0.5003 (3)	0.3319 (3)	1.2798 (2)	0.0420 (16)	
H37	0.4838	0.2721	1.2999	0.050*	
C38	0.4438 (3)	0.4167 (3)	1.31061 (19)	0.0428 (16)	
H38	0.3888	0.4148	1.3518	0.051*	
C39	0.4678 (3)	0.5043 (2)	1.2812 (2)	0.0345 (14)	
H39	0.4292	0.5622	1.3023	0.041*	
C40	0.8874 (2)	0.7819 (3)	0.72386 (17)	0.0203 (10)	
C41	0.9008 (3)	0.8327 (3)	0.76640 (15)	0.0293 (12)	
H41	0.8562	0.8410	0.8128	0.035*	
C42	0.9793 (3)	0.8713 (3)	0.74100 (19)	0.0344 (14)	
H42	0.9884	0.9061	0.7701	0.041*	
C43	1.0445 (2)	0.8591 (3)	0.67305 (19)	0.0308 (13)	
H43	1.0982	0.8856	0.6557	0.037*	
C44	1.0311 (3)	0.8083 (3)	0.63051 (15)	0.0321 (13)	
H44	1.0757	0.8000	0.5841	0.038*	
C45	0.9526 (3)	0.7696 (3)	0.65592 (16)	0.0257 (12)	
H45	0.9435	0.7349	0.6268	0.031*	
C46	0.8060 (3)	0.61942 (19)	0.72419 (16)	0.0176 (10)	
C47	0.8266 (3)	0.5212 (2)	0.77497 (12)	0.0217 (11)	
H47	0.8239	0.5126	0.8271	0.026*	
C48	0.8510 (3)	0.43569 (18)	0.74944 (15)	0.0254 (11)	
H48	0.8651	0.3686	0.7841	0.030*	
C49	0.8549 (3)	0.4483 (2)	0.67314 (17)	0.0246 (11)	
H49	0.8716	0.3899	0.6557	0.030*	
C50	0.8344 (3)	0.5465 (2)	0.62236 (12)	0.0253 (12)	
H50	0.8370	0.5551	0.5702	0.030*	
C51	0.8099 (3)	0.63205 (18)	0.64788 (14)	0.0230 (11)	
H51	0.7958	0.6992	0.6132	0.028*	
C52	0.6509 (2)	0.8450 (2)	0.72226 (17)	0.0182 (10)	
C53	0.5530 (2)	0.8515 (2)	0.76437 (14)	0.0231 (11)	
H53	0.5495	0.8038	0.8127	0.028*	
C54	0.46027 (19)	0.9277 (3)	0.73580 (17)	0.0263 (12)	
H54	0.3934	0.9321	0.7646	0.032*	
C55	0.4654 (2)	0.9974 (2)	0.66512 (18)	0.0274 (12)	
H55	0.4020	1.0495	0.6456	0.033*	
C56	0.5633 (3)	0.9910 (2)	0.62301 (14)	0.0281 (12)	
H56	0.5668	1.0387	0.5747	0.034*	
C57	0.6561 (2)	0.9148 (3)	0.65158 (16)	0.0225 (11)	
H57	0.7230	0.9104	0.6228	0.027*	
C58	0.9777 (2)	0.7831 (2)	0.41918 (18)	0.0221 (11)	
C59	1.0692 (3)	0.8022 (2)	0.39184 (17)	0.0251 (11)	
H59	1.0669	0.8654	0.3549	0.030*	
C60	1.1642 (2)	0.7287 (3)	0.41855 (19)	0.0301 (13)	
H60	1.2268	0.7417	0.3999	0.036*	
C61	1.1677 (2)	0.6362 (2)	0.47260 (19)	0.0319 (13)	
H61	1.2326	0.5860	0.4909	0.038*	
C62	1.0762 (3)	0.6172 (2)	0.49995 (16)	0.0270 (12)	
H62	1.0785	0.5540	0.5369	0.032*	
C63	0.9812 (2)	0.6906 (2)	0.47324 (17)	0.0227 (11)	
H63	0.9186	0.6776	0.4919	0.027*	
C64	0.7728 (2)	0.8193 (2)	0.41460 (17)	0.0193 (10)	
C65	0.6996 (3)	0.8321 (2)	0.47932 (16)	0.0221 (11)	
H65	0.6983	0.8731	0.5081	0.027*	
C66	0.6283 (2)	0.7847 (3)	0.50194 (15)	0.0284 (12)	
H66	0.5783	0.7935	0.5462	0.034*	
C67	0.6303 (3)	0.7246 (3)	0.45982 (19)	0.0303 (13)	
H67	0.5816	0.6923	0.4753	0.036*	
C68	0.7035 (3)	0.7119 (3)	0.39510 (19)	0.0312 (13)	
H68	0.7048	0.6708	0.3663	0.037*	
C69	0.7747 (2)	0.7592 (3)	0.37248 (16)	0.0268 (12)	
H69	0.8247	0.7505	0.3282	0.032*	
C70	0.8132 (2)	0.9726 (2)	0.43254 (17)	0.0189 (10)	
C71	0.7171 (2)	1.0564 (2)	0.41706 (17)	0.0223 (11)	
H71	0.6772	1.0576	0.3842	0.027*	
C72	0.6794 (2)	1.1384 (2)	0.44967 (19)	0.0278 (12)	
H72	0.6137	1.1957	0.4391	0.033*	
C73	0.7378 (3)	1.1367 (2)	0.49776 (18)	0.0294 (13)	
H73	0.7120	1.1928	0.5200	0.035*	
C74	0.8339 (3)	1.0529 (3)	0.51324 (17)	0.0294 (13)	
H74	0.8738	1.0517	0.5461	0.035*	
C75	0.8716 (2)	0.9708 (2)	0.48063 (18)	0.0233 (11)	
H75	0.9373	0.9135	0.4912	0.028*	
C76	0.8834 (3)	0.9200 (2)	0.29298 (12)	0.0216 (11)	
C77	0.9541 (3)	0.84763 (19)	0.25304 (17)	0.0276 (12)	
H77	0.9879	0.7763	0.2792	0.033*	
C78	0.9755 (3)	0.8796 (2)	0.17476 (17)	0.0329 (14)	
H78	1.0238	0.8301	0.1475	0.040*	
C79	0.9261 (3)	0.9839 (3)	0.13641 (12)	0.0314 (13)	
H79	0.9407	1.0058	0.0829	0.038*	
C80	0.8553 (3)	1.05630 (19)	0.17636 (16)	0.0267 (12)	
H80	0.8216	1.1276	0.1502	0.032*	
C81	0.8340 (2)	1.0244 (2)	0.25464 (16)	0.0210 (11)	
H81	0.7856	1.0738	0.2819	0.025*	

### Atomic displacement parameters (Å^2^)

**Table:**

	*U*^11^	*U*^22^	*U*^33^	*U*^12^	*U*^13^	*U*^23^
Sn1	0.01473 (17)	0.01544 (17)	0.01310 (16)	−0.00479 (13)	−0.00460 (12)	−0.00301 (13)
As1	0.0186 (3)	0.0259 (3)	0.0140 (3)	−0.0083 (2)	−0.0023 (2)	−0.0063 (2)
As2	0.0163 (3)	0.0201 (3)	0.0120 (2)	−0.0081 (2)	−0.00233 (19)	−0.0025 (2)
O1	0.030 (2)	0.041 (2)	0.0141 (18)	−0.0184 (19)	−0.0009 (15)	−0.0061 (17)
O2	0.025 (2)	0.031 (2)	0.0131 (17)	−0.0127 (17)	−0.0052 (15)	0.0002 (15)
B1	0.028 (3)	0.021 (3)	0.018 (3)	−0.010 (3)	−0.007 (2)	−0.005 (2)
C1	0.030 (3)	0.022 (3)	0.028 (3)	−0.007 (2)	−0.009 (2)	−0.008 (2)
C2	0.026 (3)	0.016 (3)	0.033 (3)	−0.006 (2)	−0.007 (2)	−0.006 (2)
C3	0.021 (3)	0.015 (3)	0.037 (3)	−0.001 (2)	−0.011 (2)	−0.005 (2)
C4	0.035 (3)	0.024 (3)	0.033 (3)	−0.012 (3)	−0.001 (3)	−0.013 (2)
C5	0.037 (3)	0.031 (3)	0.034 (3)	−0.017 (3)	−0.009 (3)	−0.014 (3)
C6	0.026 (3)	0.033 (3)	0.047 (4)	−0.011 (3)	−0.009 (3)	−0.019 (3)
C7	0.026 (3)	0.026 (3)	0.033 (3)	−0.009 (2)	−0.001 (2)	−0.013 (2)
C8	0.017 (3)	0.025 (3)	0.018 (2)	−0.011 (2)	−0.006 (2)	−0.002 (2)
C9	0.018 (3)	0.021 (3)	0.019 (3)	−0.003 (2)	−0.008 (2)	−0.002 (2)
C10	0.022 (3)	0.031 (3)	0.023 (3)	−0.003 (2)	−0.002 (2)	−0.003 (2)
C11	0.034 (3)	0.024 (3)	0.036 (3)	−0.008 (3)	0.000 (3)	0.001 (3)
C12	0.028 (3)	0.022 (3)	0.040 (3)	−0.010 (2)	−0.003 (3)	−0.003 (3)
C13	0.023 (3)	0.030 (3)	0.032 (3)	−0.008 (2)	−0.005 (2)	−0.014 (2)
C14	0.022 (3)	0.023 (3)	0.020 (3)	−0.005 (2)	−0.009 (2)	−0.002 (2)
C15	0.020 (3)	0.024 (3)	0.020 (3)	−0.012 (2)	−0.002 (2)	−0.006 (2)
C16	0.021 (3)	0.030 (3)	0.020 (3)	−0.015 (2)	−0.004 (2)	−0.008 (2)
C17	0.039 (3)	0.039 (3)	0.026 (3)	−0.026 (3)	0.007 (3)	−0.012 (3)
C18	0.044 (4)	0.055 (4)	0.045 (4)	−0.038 (4)	0.019 (3)	−0.019 (3)
C19	0.020 (3)	0.072 (5)	0.059 (4)	−0.026 (3)	0.009 (3)	−0.036 (4)
C20	0.022 (3)	0.047 (4)	0.043 (4)	−0.007 (3)	−0.011 (3)	−0.012 (3)
C21	0.031 (3)	0.042 (3)	0.021 (3)	−0.017 (3)	−0.004 (2)	−0.009 (3)
C22	0.024 (3)	0.024 (3)	0.021 (3)	−0.012 (2)	0.002 (2)	−0.006 (2)
C23	0.035 (3)	0.034 (3)	0.029 (3)	−0.020 (3)	0.003 (2)	−0.012 (3)
C24	0.050 (4)	0.039 (4)	0.042 (4)	−0.015 (3)	0.007 (3)	−0.023 (3)
C25	0.055 (5)	0.038 (4)	0.053 (4)	−0.012 (4)	0.013 (4)	−0.018 (3)
C26	0.027 (4)	0.049 (4)	0.064 (5)	0.000 (3)	−0.005 (3)	−0.011 (4)
C27	0.037 (4)	0.049 (4)	0.035 (4)	−0.014 (3)	−0.005 (3)	−0.013 (3)
C28	0.023 (3)	0.025 (3)	0.019 (3)	−0.009 (2)	−0.003 (2)	−0.007 (2)
C29	0.033 (3)	0.028 (3)	0.025 (3)	−0.014 (3)	−0.006 (2)	−0.007 (2)
C30	0.026 (3)	0.038 (3)	0.027 (3)	0.000 (3)	−0.016 (2)	−0.007 (3)
C31	0.017 (3)	0.043 (3)	0.029 (3)	−0.006 (3)	−0.006 (2)	−0.014 (3)
C32	0.029 (3)	0.046 (4)	0.022 (3)	−0.021 (3)	−0.002 (2)	−0.011 (3)
C33	0.031 (3)	0.024 (3)	0.016 (2)	−0.012 (2)	−0.002 (2)	−0.005 (2)
C34	0.022 (3)	0.027 (3)	0.020 (3)	−0.008 (2)	−0.006 (2)	−0.005 (2)
C35	0.037 (4)	0.036 (3)	0.037 (3)	−0.015 (3)	0.003 (3)	−0.014 (3)
C36	0.051 (4)	0.030 (3)	0.053 (4)	−0.014 (3)	−0.008 (3)	−0.015 (3)
C37	0.051 (4)	0.037 (4)	0.045 (4)	−0.026 (3)	−0.009 (3)	−0.004 (3)
C38	0.045 (4)	0.045 (4)	0.038 (4)	−0.026 (3)	0.005 (3)	−0.009 (3)
C39	0.034 (3)	0.041 (4)	0.034 (3)	−0.021 (3)	0.006 (3)	−0.016 (3)
C40	0.020 (3)	0.023 (3)	0.016 (2)	−0.009 (2)	−0.006 (2)	0.000 (2)
C41	0.032 (3)	0.034 (3)	0.024 (3)	−0.016 (3)	0.001 (2)	−0.009 (2)
C42	0.037 (4)	0.039 (3)	0.038 (3)	−0.024 (3)	−0.013 (3)	−0.004 (3)
C43	0.029 (3)	0.030 (3)	0.031 (3)	−0.019 (3)	−0.015 (3)	0.011 (2)
C44	0.033 (3)	0.037 (3)	0.024 (3)	−0.022 (3)	−0.002 (2)	0.004 (2)
C45	0.024 (3)	0.034 (3)	0.021 (3)	−0.016 (2)	−0.002 (2)	−0.004 (2)
C46	0.020 (3)	0.012 (2)	0.021 (2)	−0.004 (2)	−0.011 (2)	−0.0016 (19)
C47	0.021 (3)	0.027 (3)	0.016 (2)	−0.010 (2)	−0.005 (2)	−0.002 (2)
C48	0.027 (3)	0.021 (3)	0.027 (3)	−0.012 (2)	−0.004 (2)	−0.002 (2)
C49	0.026 (3)	0.024 (3)	0.025 (3)	−0.011 (2)	−0.001 (2)	−0.009 (2)
C50	0.031 (3)	0.028 (3)	0.019 (3)	−0.013 (2)	−0.003 (2)	−0.006 (2)
C51	0.029 (3)	0.024 (3)	0.013 (2)	−0.012 (2)	0.000 (2)	−0.003 (2)
C52	0.020 (3)	0.016 (2)	0.020 (2)	−0.004 (2)	−0.009 (2)	−0.006 (2)
C53	0.023 (3)	0.022 (3)	0.018 (3)	−0.005 (2)	−0.006 (2)	−0.002 (2)
C54	0.015 (3)	0.034 (3)	0.028 (3)	−0.007 (2)	−0.002 (2)	−0.012 (2)
C55	0.035 (3)	0.017 (3)	0.031 (3)	−0.002 (2)	−0.022 (3)	−0.006 (2)
C56	0.033 (3)	0.026 (3)	0.021 (3)	−0.013 (3)	−0.006 (2)	0.002 (2)
C57	0.020 (3)	0.023 (3)	0.018 (3)	−0.008 (2)	−0.003 (2)	−0.001 (2)
C58	0.029 (3)	0.022 (3)	0.016 (2)	−0.008 (2)	−0.003 (2)	−0.008 (2)
C59	0.019 (3)	0.028 (3)	0.031 (3)	−0.011 (2)	−0.004 (2)	−0.010 (2)
C60	0.024 (3)	0.038 (3)	0.037 (3)	−0.013 (3)	−0.011 (2)	−0.014 (3)
C61	0.030 (3)	0.035 (3)	0.028 (3)	−0.002 (3)	−0.016 (3)	−0.010 (3)
C62	0.037 (3)	0.030 (3)	0.012 (2)	−0.011 (3)	−0.008 (2)	−0.002 (2)
C63	0.022 (3)	0.028 (3)	0.018 (3)	−0.009 (2)	−0.003 (2)	−0.007 (2)
C64	0.014 (2)	0.019 (2)	0.022 (3)	−0.002 (2)	−0.010 (2)	−0.003 (2)
C65	0.027 (3)	0.023 (3)	0.015 (2)	−0.011 (2)	−0.008 (2)	0.002 (2)
C66	0.036 (3)	0.034 (3)	0.019 (3)	−0.020 (3)	−0.006 (2)	0.000 (2)
C67	0.026 (3)	0.028 (3)	0.040 (3)	−0.017 (3)	−0.009 (3)	−0.002 (3)
C68	0.032 (3)	0.034 (3)	0.038 (3)	−0.015 (3)	−0.005 (3)	−0.019 (3)
C69	0.018 (3)	0.029 (3)	0.037 (3)	−0.009 (2)	−0.002 (2)	−0.015 (3)
C70	0.023 (3)	0.023 (3)	0.017 (2)	−0.017 (2)	−0.003 (2)	−0.002 (2)
C71	0.022 (3)	0.030 (3)	0.023 (3)	−0.016 (2)	0.000 (2)	−0.011 (2)
C72	0.028 (3)	0.026 (3)	0.027 (3)	−0.009 (2)	−0.001 (2)	−0.009 (2)
C73	0.035 (3)	0.038 (3)	0.027 (3)	−0.025 (3)	0.013 (2)	−0.020 (3)
C74	0.039 (3)	0.035 (3)	0.023 (3)	−0.024 (3)	0.001 (2)	−0.008 (2)
C75	0.022 (3)	0.032 (3)	0.022 (3)	−0.018 (2)	−0.003 (2)	−0.006 (2)
C76	0.015 (3)	0.026 (3)	0.021 (3)	−0.003 (2)	−0.004 (2)	−0.010 (2)
C77	0.028 (3)	0.025 (3)	0.022 (3)	−0.005 (2)	−0.005 (2)	−0.005 (2)
C78	0.031 (3)	0.045 (4)	0.020 (3)	−0.010 (3)	−0.001 (2)	−0.014 (3)
C79	0.031 (3)	0.051 (4)	0.014 (3)	−0.023 (3)	−0.002 (2)	−0.003 (3)
C80	0.037 (3)	0.028 (3)	0.023 (3)	−0.019 (3)	−0.010 (2)	−0.002 (2)
C81	0.025 (3)	0.019 (3)	0.021 (3)	−0.008 (2)	−0.007 (2)	−0.005 (2)

### Geometric parameters (Å, °)

**Table:**

Sn1—C1	2.150 (5)	C36—C37	1.3900
Sn1—C8	2.145 (5)	C36—H36	0.9500
Sn1—C15	2.144 (5)	C37—C38	1.3900
Sn1—O1	2.175 (3)	C37—H37	0.9500
Sn1—O2	2.225 (3)	C38—C39	1.3900
As1—O1	1.657 (3)	C38—H38	0.9500
As1—C22	1.899 (3)	C39—H39	0.9500
As1—C34	1.909 (3)	C40—C41	1.3900
As1—C28	1.909 (2)	C40—C45	1.3900
As2—O2	1.651 (3)	C41—C42	1.3900
As2—C40	1.893 (2)	C41—H41	0.9500
As2—C52	1.904 (2)	C42—C43	1.3900
As2—C46	1.909 (2)	C42—H42	0.9500
B1—C58	1.695 (6)	C43—C44	1.3900
B1—C70	1.697 (6)	C43—H43	0.9500
B1—C64	1.701 (6)	C44—C45	1.3900
B1—C76	1.712 (6)	C44—H44	0.9500
C1—C2	1.511 (5)	C45—H45	0.9500
C1—H1A	0.9900	C46—C47	1.3900
C1—H1B	0.9900	C46—C51	1.3900
C2—C3	1.3900	C47—C48	1.3900
C2—C7	1.3900	C47—H47	0.9500
C3—C4	1.3900	C48—C49	1.3900
C3—H3	0.9500	C48—H48	0.9500
C4—C5	1.3900	C49—C50	1.3900
C4—H4	0.9500	C49—H49	0.9500
C5—C6	1.3900	C50—C51	1.3900
C5—H5	0.9500	C50—H50	0.9500
C6—C7	1.3900	C51—H51	0.9500
C6—H6	0.9500	C52—C53	1.3900
C7—H7	0.9500	C52—C57	1.3900
C8—C9	1.531 (5)	C53—C54	1.3900
C8—H8A	0.9900	C53—H53	0.9500
C8—H8B	0.9900	C54—C55	1.3900
C9—C10	1.3900	C54—H54	0.9500
C9—C14	1.3900	C55—C56	1.3900
C10—C11	1.3900	C55—H55	0.9500
C10—H10	0.9500	C56—C57	1.3900
C11—C12	1.3900	C56—H56	0.9500
C11—H11	0.9500	C57—H57	0.9500
C12—C13	1.3900	C58—C59	1.3900
C12—H12	0.9500	C58—C63	1.3900
C13—C14	1.3900	C59—C60	1.3900
C13—H13	0.9500	C59—H59	0.9500
C14—H14	0.9500	C60—C61	1.3900
C15—C16	1.520 (5)	C60—H60	0.9500
C15—H15A	0.9900	C61—C62	1.3900
C15—H15B	0.9900	C61—H61	0.9500
C16—C17	1.3900	C62—C63	1.3900
C16—C21	1.3900	C62—H62	0.9500
C17—C18	1.3900	C63—H63	0.9500
C17—H17	0.9500	C64—C65	1.3900
C18—C19	1.3900	C64—C69	1.3900
C18—H18	0.9500	C65—C66	1.3900
C19—C20	1.3900	C65—H65	0.9500
C19—H19	0.9500	C66—C67	1.3900
C20—C21	1.3900	C66—H66	0.9500
C20—H20	0.9500	C67—C68	1.3900
C21—H21	0.9500	C67—H67	0.9500
C22—C23	1.3900	C68—C69	1.3900
C22—C27	1.3900	C68—H68	0.9500
C23—C24	1.3900	C69—H69	0.9500
C23—H23	0.9500	C70—C71	1.3900
C24—C25	1.3900	C70—C75	1.3900
C24—H24	0.9500	C71—C72	1.3900
C25—C26	1.3900	C71—H71	0.9500
C25—H25	0.9500	C72—C73	1.3900
C26—C27	1.3900	C72—H72	0.9500
C26—H26	0.9500	C73—C74	1.3900
C27—H27	0.9500	C73—H73	0.9500
C28—C29	1.3900	C74—C75	1.3900
C28—C33	1.3900	C74—H74	0.9500
C29—C30	1.3900	C75—H75	0.9500
C29—H29	0.9500	C76—C77	1.3900
C30—C31	1.3900	C76—C81	1.3900
C30—H30	0.9500	C77—C78	1.3900
C31—C32	1.3900	C77—H77	0.9500
C31—H31	0.9500	C78—C79	1.3900
C32—C33	1.3900	C78—H78	0.9500
C32—H32	0.9500	C79—C80	1.3900
C33—H33	0.9500	C79—H79	0.9500
C34—C35	1.3900	C80—C81	1.3900
C34—C39	1.3900	C80—H80	0.9500
C35—C36	1.3900	C81—H81	0.9500
C35—H35	0.9500		
			
C1—Sn1—C8	114.4 (2)	C34—C35—C36	120.0
C1—Sn1—C15	119.7 (2)	C34—C35—H35	120.0
C8—Sn1—C15	125.9 (2)	C36—C35—H35	120.0
C15—Sn1—O1	86.24 (16)	C35—C36—C37	120.0
C8—Sn1—O1	93.12 (16)	C35—C36—H36	120.0
C1—Sn1—O1	93.11 (18)	C37—C36—H36	120.0
C15—Sn1—O2	88.98 (16)	C38—C37—C36	120.0
C8—Sn1—O2	85.65 (16)	C38—C37—H37	120.0
C1—Sn1—O2	93.57 (17)	C36—C37—H37	120.0
O1—Sn1—O2	173.1 (1)	C37—C38—C39	120.0
O1—As1—C22	109.92 (17)	C37—C38—H38	120.0
O1—As1—C34	111.14 (16)	C39—C38—H38	120.0
C22—As1—C34	107.37 (16)	C38—C39—C34	120.0
O1—As1—C28	110.98 (16)	C38—C39—H39	120.0
C22—As1—C28	109.80 (15)	C34—C39—H39	120.0
C34—As1—C28	107.54 (15)	C41—C40—C45	120.0
O2—As2—C40	109.27 (16)	C41—C40—As2	117.73 (17)
O2—As2—C52	111.94 (16)	C45—C40—As2	122.25 (17)
C40—As2—C52	108.31 (14)	C40—C41—C42	120.0
O2—As2—C46	109.16 (15)	C40—C41—H41	120.0
C40—As2—C46	111.20 (14)	C42—C41—H41	120.0
C52—As2—C46	106.96 (14)	C43—C42—C41	120.0
As1—O1—Sn1	165.3 (2)	C43—C42—H42	120.0
As2—O2—Sn1	160.3 (2)	C41—C42—H42	120.0
C58—B1—C70	109.7 (3)	C44—C43—C42	120.0
C58—B1—C64	108.3 (4)	C44—C43—H43	120.0
C70—B1—C64	108.3 (4)	C42—C43—H43	120.0
C58—B1—C76	107.9 (4)	C43—C44—C45	120.0
C70—B1—C76	112.3 (4)	C43—C44—H44	120.0
C64—B1—C76	110.3 (3)	C45—C44—H44	120.0
C2—C1—Sn1	112.2 (3)	C44—C45—C40	120.0
C2—C1—H1A	109.2	C44—C45—H45	120.0
Sn1—C1—H1A	109.2	C40—C45—H45	120.0
C2—C1—H1B	109.2	C47—C46—C51	120.0
Sn1—C1—H1B	109.2	C47—C46—As2	119.09 (16)
H1A—C1—H1B	107.9	C51—C46—As2	120.89 (16)
C3—C2—C7	120.0	C46—C47—C48	120.0
C3—C2—C1	119.5 (3)	C46—C47—H47	120.0
C7—C2—C1	120.5 (3)	C48—C47—H47	120.0
C4—C3—C2	120.0	C49—C48—C47	120.0
C4—C3—H3	120.0	C49—C48—H48	120.0
C2—C3—H3	120.0	C47—C48—H48	120.0
C3—C4—C5	120.0	C50—C49—C48	120.0
C3—C4—H4	120.0	C50—C49—H49	120.0
C5—C4—H4	120.0	C48—C49—H49	120.0
C4—C5—C6	120.0	C49—C50—C51	120.0
C4—C5—H5	120.0	C49—C50—H50	120.0
C6—C5—H5	120.0	C51—C50—H50	120.0
C7—C6—C5	120.0	C50—C51—C46	120.0
C7—C6—H6	120.0	C50—C51—H51	120.0
C5—C6—H6	120.0	C46—C51—H51	120.0
C6—C7—C2	120.0	C53—C52—C57	120.0
C6—C7—H7	120.0	C53—C52—As2	118.93 (17)
C2—C7—H7	120.0	C57—C52—As2	120.97 (17)
C9—C8—Sn1	112.6 (3)	C52—C53—C54	120.0
C9—C8—H8A	109.1	C52—C53—H53	120.0
Sn1—C8—H8A	109.1	C54—C53—H53	120.0
C9—C8—H8B	109.1	C53—C54—C55	120.0
Sn1—C8—H8B	109.1	C53—C54—H54	120.0
H8A—C8—H8B	107.8	C55—C54—H54	120.0
C10—C9—C14	120.0	C56—C55—C54	120.0
C10—C9—C8	120.6 (3)	C56—C55—H55	120.0
C14—C9—C8	119.3 (3)	C54—C55—H55	120.0
C11—C10—C9	120.0	C57—C56—C55	120.0
C11—C10—H10	120.0	C57—C56—H56	120.0
C9—C10—H10	120.0	C55—C56—H56	120.0
C10—C11—C12	120.0	C56—C57—C52	120.0
C10—C11—H11	120.0	C56—C57—H57	120.0
C12—C11—H11	120.0	C52—C57—H57	120.0
C13—C12—C11	120.0	C59—C58—C63	120.0
C13—C12—H12	120.0	C59—C58—B1	119.7 (3)
C11—C12—H12	120.0	C63—C58—B1	120.3 (3)
C12—C13—C14	120.0	C60—C59—C58	120.0
C12—C13—H13	120.0	C60—C59—H59	120.0
C14—C13—H13	120.0	C58—C59—H59	120.0
C13—C14—C9	120.0	C59—C60—C61	120.0
C13—C14—H14	120.0	C59—C60—H60	120.0
C9—C14—H14	120.0	C61—C60—H60	120.0
C16—C15—Sn1	113.7 (3)	C62—C61—C60	120.0
C16—C15—H15A	108.8	C62—C61—H61	120.0
Sn1—C15—H15A	108.8	C60—C61—H61	120.0
C16—C15—H15B	108.8	C61—C62—C63	120.0
Sn1—C15—H15B	108.8	C61—C62—H62	120.0
H15A—C15—H15B	107.7	C63—C62—H62	120.0
C17—C16—C21	120.0	C62—C63—C58	120.0
C17—C16—C15	118.9 (3)	C62—C63—H63	120.0
C21—C16—C15	121.1 (3)	C58—C63—H63	120.0
C18—C17—C16	120.0	C65—C64—C69	120.0
C18—C17—H17	120.0	C65—C64—B1	119.1 (3)
C16—C17—H17	120.0	C69—C64—B1	120.9 (3)
C17—C18—C19	120.0	C66—C65—C64	120.0
C17—C18—H18	120.0	C66—C65—H65	120.0
C19—C18—H18	120.0	C64—C65—H65	120.0
C20—C19—C18	120.0	C65—C66—C67	120.0
C20—C19—H19	120.0	C65—C66—H66	120.0
C18—C19—H19	120.0	C67—C66—H66	120.0
C21—C20—C19	120.0	C68—C67—C66	120.0
C21—C20—H20	120.0	C68—C67—H67	120.0
C19—C20—H20	120.0	C66—C67—H67	120.0
C20—C21—C16	120.0	C67—C68—C69	120.0
C20—C21—H21	120.0	C67—C68—H68	120.0
C16—C21—H21	120.0	C69—C68—H68	120.0
C23—C22—C27	120.0	C68—C69—C64	120.0
C23—C22—As1	120.4 (2)	C68—C69—H69	120.0
C27—C22—As1	119.5 (2)	C64—C69—H69	120.0
C22—C23—C24	120.0	C71—C70—C75	120.0
C22—C23—H23	120.0	C71—C70—B1	119.6 (3)
C24—C23—H23	120.0	C75—C70—B1	120.4 (3)
C23—C24—C25	120.0	C72—C71—C70	120.0
C23—C24—H24	120.0	C72—C71—H71	120.0
C25—C24—H24	120.0	C70—C71—H71	120.0
C26—C25—C24	120.0	C71—C72—C73	120.0
C26—C25—H25	120.0	C71—C72—H72	120.0
C24—C25—H25	120.0	C73—C72—H72	120.0
C25—C26—C27	120.0	C72—C73—C74	120.0
C25—C26—H26	120.0	C72—C73—H73	120.0
C27—C26—H26	120.0	C74—C73—H73	120.0
C26—C27—C22	120.0	C75—C74—C73	120.0
C26—C27—H27	120.0	C75—C74—H74	120.0
C22—C27—H27	120.0	C73—C74—H74	120.0
C29—C28—C33	120.0	C74—C75—C70	120.0
C29—C28—As1	120.76 (18)	C74—C75—H75	120.0
C33—C28—As1	119.23 (18)	C70—C75—H75	120.0
C30—C29—C28	120.0	C77—C76—C81	120.0
C30—C29—H29	120.0	C77—C76—B1	117.6 (3)
C28—C29—H29	120.0	C81—C76—B1	122.4 (3)
C29—C30—C31	120.0	C76—C77—C78	120.0
C29—C30—H30	120.0	C76—C77—H77	120.0
C31—C30—H30	120.0	C78—C77—H77	120.0
C32—C31—C30	120.0	C77—C78—C79	120.0
C32—C31—H31	120.0	C77—C78—H78	120.0
C30—C31—H31	120.0	C79—C78—H78	120.0
C31—C32—C33	120.0	C78—C79—C80	120.0
C31—C32—H32	120.0	C78—C79—H79	120.0
C33—C32—H32	120.0	C80—C79—H79	120.0
C32—C33—C28	120.0	C81—C80—C79	120.0
C32—C33—H33	120.0	C81—C80—H80	120.0
C28—C33—H33	120.0	C79—C80—H80	120.0
C35—C34—C39	120.0	C80—C81—C76	120.0
C35—C34—As1	119.74 (19)	C80—C81—H81	120.0
C39—C34—As1	120.18 (19)	C76—C81—H81	120.0
			
C22—As1—O1—Sn1	126.5 (9)	C52—As2—C40—C41	88.5 (2)
C34—As1—O1—Sn1	−114.8 (9)	C46—As2—C40—C41	−154.27 (18)
C28—As1—O1—Sn1	4.8 (9)	O2—As2—C40—C45	148.1 (2)
C15—Sn1—O1—As1	139.5 (9)	C52—As2—C40—C45	−89.7 (2)
C8—Sn1—O1—As1	13.6 (9)	C46—As2—C40—C45	27.6 (2)
C1—Sn1—O1—As1	−101.0 (9)	C45—C40—C41—C42	0.0
C40—As2—O2—Sn1	128.4 (6)	As2—C40—C41—C42	−178.2 (2)
C52—As2—O2—Sn1	8.4 (7)	C40—C41—C42—C43	0.0
C46—As2—O2—Sn1	−109.8 (6)	C41—C42—C43—C44	0.0
C15—Sn1—O2—As2	62.7 (6)	C42—C43—C44—C45	0.0
C8—Sn1—O2—As2	−171.2 (6)	C43—C44—C45—C40	0.0
C1—Sn1—O2—As2	−57.0 (6)	C41—C40—C45—C44	0.0
C15—Sn1—C1—C2	144.6 (3)	As2—C40—C45—C44	178.1 (3)
C8—Sn1—C1—C2	−37.7 (4)	O2—As2—C46—C47	−9.3 (2)
O1—Sn1—C1—C2	57.2 (4)	C40—As2—C46—C47	111.3 (2)
O2—Sn1—C1—C2	−124.5 (3)	C52—As2—C46—C47	−130.62 (19)
Sn1—C1—C2—C3	−100.8 (3)	O2—As2—C46—C51	172.3 (2)
Sn1—C1—C2—C7	77.3 (4)	C40—As2—C46—C51	−67.0 (2)
C7—C2—C3—C4	0.0	C52—As2—C46—C51	51.0 (2)
C1—C2—C3—C4	178.1 (4)	C51—C46—C47—C48	0.0
C2—C3—C4—C5	0.0	As2—C46—C47—C48	−178.4 (2)
C3—C4—C5—C6	0.0	C46—C47—C48—C49	0.0
C4—C5—C6—C7	0.0	C47—C48—C49—C50	0.0
C5—C6—C7—C2	0.0	C48—C49—C50—C51	0.0
C3—C2—C7—C6	0.0	C49—C50—C51—C46	0.0
C1—C2—C7—C6	−178.1 (4)	C47—C46—C51—C50	0.0
C15—Sn1—C8—C9	−4.9 (4)	As2—C46—C51—C50	178.3 (2)
C1—Sn1—C8—C9	177.5 (3)	O2—As2—C52—C53	−30.3 (2)
O1—Sn1—C8—C9	82.7 (3)	C40—As2—C52—C53	−150.86 (18)
O2—Sn1—C8—C9	−90.5 (3)	C46—As2—C52—C53	89.19 (19)
Sn1—C8—C9—C10	−91.8 (3)	O2—As2—C52—C57	153.30 (19)
Sn1—C8—C9—C14	85.1 (3)	C40—As2—C52—C57	32.8 (2)
C14—C9—C10—C11	0.0	C46—As2—C52—C57	−87.2 (2)
C8—C9—C10—C11	176.9 (4)	C57—C52—C53—C54	0.0
C9—C10—C11—C12	0.0	As2—C52—C53—C54	−176.4 (2)
C10—C11—C12—C13	0.0	C52—C53—C54—C55	0.0
C11—C12—C13—C14	0.0	C53—C54—C55—C56	0.0
C12—C13—C14—C9	0.0	C54—C55—C56—C57	0.0
C10—C9—C14—C13	0.0	C55—C56—C57—C52	0.0
C8—C9—C14—C13	−176.9 (3)	C53—C52—C57—C56	0.0
C8—Sn1—C15—C16	138.9 (3)	As2—C52—C57—C56	176.3 (2)
C1—Sn1—C15—C16	−43.7 (4)	C70—B1—C58—C59	−75.9 (4)
O1—Sn1—C15—C16	47.7 (3)	C64—B1—C58—C59	166.1 (2)
O2—Sn1—C15—C16	−137.2 (3)	C76—B1—C58—C59	46.7 (4)
Sn1—C15—C16—C17	−112.3 (3)	C70—B1—C58—C63	101.3 (4)
Sn1—C15—C16—C21	67.6 (4)	C64—B1—C58—C63	−16.7 (4)
C21—C16—C17—C18	0.0	C76—B1—C58—C63	−136.1 (3)
C15—C16—C17—C18	179.9 (3)	C63—C58—C59—C60	0.0
C16—C17—C18—C19	0.0	B1—C58—C59—C60	177.2 (3)
C17—C18—C19—C20	0.0	C58—C59—C60—C61	0.0
C18—C19—C20—C21	0.0	C59—C60—C61—C62	0.0
C19—C20—C21—C16	0.0	C60—C61—C62—C63	0.0
C17—C16—C21—C20	0.0	C61—C62—C63—C58	0.0
C15—C16—C21—C20	−179.9 (3)	C59—C58—C63—C62	0.0
O1—As1—C22—C23	−136.7 (2)	B1—C58—C63—C62	−177.1 (3)
C34—As1—C22—C23	102.3 (2)	C58—B1—C64—C65	98.9 (3)
C28—As1—C22—C23	−14.4 (3)	C70—B1—C64—C65	−20.0 (4)
O1—As1—C22—C27	45.6 (3)	C76—B1—C64—C65	−143.3 (3)
C34—As1—C22—C27	−75.4 (2)	C58—B1—C64—C69	−79.6 (4)
C28—As1—C22—C27	167.9 (2)	C70—B1—C64—C69	161.5 (3)
C27—C22—C23—C24	0.0	C76—B1—C64—C69	38.2 (4)
As1—C22—C23—C24	−177.7 (3)	C69—C64—C65—C66	0.0
C22—C23—C24—C25	0.0	B1—C64—C65—C66	−178.5 (3)
C23—C24—C25—C26	0.0	C64—C65—C66—C67	0.0
C24—C25—C26—C27	0.0	C65—C66—C67—C68	0.0
C25—C26—C27—C22	0.0	C66—C67—C68—C69	0.0
C23—C22—C27—C26	0.0	C67—C68—C69—C64	0.0
As1—C22—C27—C26	177.7 (3)	C65—C64—C69—C68	0.0
O1—As1—C28—C29	−135.3 (2)	B1—C64—C69—C68	178.5 (3)
C22—As1—C28—C29	103.0 (2)	C58—B1—C70—C71	−179.2 (3)
C34—As1—C28—C29	−13.5 (2)	C64—B1—C70—C71	−61.2 (4)
O1—As1—C28—C33	43.5 (2)	C76—B1—C70—C71	60.9 (4)
C22—As1—C28—C33	−78.2 (2)	C58—B1—C70—C75	3.6 (5)
C34—As1—C28—C33	165.30 (19)	C64—B1—C70—C75	121.6 (3)
C33—C28—C29—C30	0.0	C76—B1—C70—C75	−116.3 (3)
As1—C28—C29—C30	178.8 (2)	C75—C70—C71—C72	0.0
C28—C29—C30—C31	0.0	B1—C70—C71—C72	−177.2 (3)
C29—C30—C31—C32	0.0	C70—C71—C72—C73	0.0
C30—C31—C32—C33	0.0	C71—C72—C73—C74	0.0
C31—C32—C33—C28	0.0	C72—C73—C74—C75	0.0
C29—C28—C33—C32	0.0	C73—C74—C75—C70	0.0
As1—C28—C33—C32	−178.8 (2)	C71—C70—C75—C74	0.0
O1—As1—C34—C35	43.5 (3)	B1—C70—C75—C74	177.2 (3)
C22—As1—C34—C35	163.8 (2)	C58—B1—C76—C77	37.5 (4)
C28—As1—C34—C35	−78.1 (2)	C70—B1—C76—C77	158.5 (3)
O1—As1—C34—C39	−139.7 (2)	C64—B1—C76—C77	−80.6 (4)
C22—As1—C34—C39	−19.5 (3)	C58—B1—C76—C81	−142.3 (3)
C28—As1—C34—C39	98.6 (2)	C70—B1—C76—C81	−21.3 (5)
C39—C34—C35—C36	0.0	C64—B1—C76—C81	99.7 (4)
As1—C34—C35—C36	176.7 (3)	C81—C76—C77—C78	0.0
C34—C35—C36—C37	0.0	B1—C76—C77—C78	−179.7 (4)
C35—C36—C37—C38	0.0	C76—C77—C78—C79	0.0
C36—C37—C38—C39	0.0	C77—C78—C79—C80	0.0
C37—C38—C39—C34	0.0	C78—C79—C80—C81	0.0
C35—C34—C39—C38	0.0	C79—C80—C81—C76	0.0
As1—C34—C39—C38	−176.7 (3)	C77—C76—C81—C80	0.0
O2—As2—C40—C41	−33.7 (2)	B1—C76—C81—C80	179.7 (4)
